# An Exceptionally Rare Primary Epithelioid Rhabdomyosarcomas of the Stomach: A Case Report

**DOI:** 10.7759/cureus.26046

**Published:** 2022-06-17

**Authors:** Lav Kumar Shah, Nashruva Jahan Mony, Sumitanand Mishra, Biswas Pant

**Affiliations:** 1 Surgery, Patan Academy of Health Sciences, Lalitpur, NPL; 2 Surgery, Nepal Korea Friendship Municipality Hospital (NKFMH), Madhyapur Thimi, NPL; 3 Medicine, Nepal Korea Friendship Municipality Hospital, Bhaktapur, NPL; 4 Radiology, Himal Hospital, Kathmandu, NPL; 5 Internal Medicine, Patan Academy of Health Science, Kathmandu, NPL

**Keywords:** cytokeratin, rhabdomyosarcomas, fibrin, desmin, epithelioid cell, myoepithelial cell, immunohistochemistry, billroth ii reconstruction, gastric outlet obstruction, primary epithelioid rhabdomyosarcomas of stomach

## Abstract

Rhabdomyosarcoma (RMS) is a common soft tissue tumor in adults, but RMS's causes and risk factors are unknown. We present a case of a 62-year-old man with RMS who presented with feelings of fullness after meals and vomiting for the previous five months with anorexia and weight loss for four months. He reported feeling a rolling mass in his belly that moves from left to right. He was initially diagnosed with gastric outlet obstruction due to stomach carcinoma. During the surgical operation, we noted the gross appearance was unlike typical adenocarcinoma or lymphoma of the stomach. Histopathological evaluation of the specimen confirmed a diagnosis of primary epithelial RMS of the stomach. When treating RMS, expertise in immunohistochemistry, molecular biology, genetics, or ultrastructure may be necessary. Information on the appropriate laboratory investigations and management protocol is limited, but an early diagnosis can change the course of treatment and improve patient outcomes.

## Introduction

There are several different types of adult soft-tissue sarcoma, which are thought to originate from a basic mesenchymal cell. Tumors that look like a muscle, fibrous connective tissue, or fat can develop in various anatomical places. The incidence of these tumors is estimated to be five per million in the United States [1,2], comprising just 3% to 4% of all pediatric malignancies. The incidence of these tumors is extremely low in the general population. Although the genitourinary system and extremities are common sites, rhabdomyosarcoma (RMS) of the stomach is the most typical presentation. We present a case of a man with RMS initially diagnosed as gastric outlet obstruction due to stomach carcinoma.

## Case presentation

**Figure 1:**
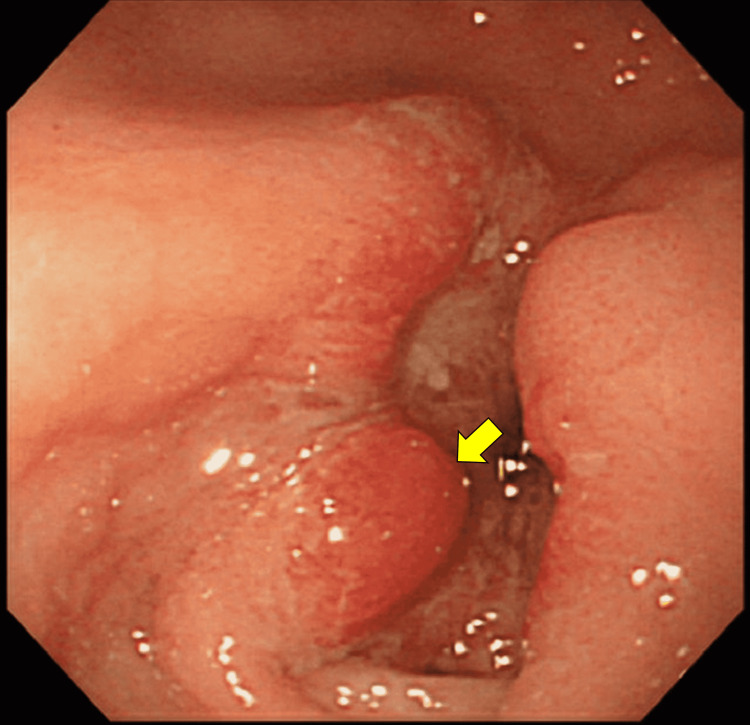
Endoscopy showing an ulcerated mass in the stomach suggestive of carcinoma

A 62-year-old man reported to the surgery outpatient department reporting feelings of fullness after meals and vomiting for the previous five months. For the past four months, he has experienced anorexia and weight loss. The sense of fullness after a meal occurs later in the evening, and he reported concerns about a rolling mass in his belly that moves from left to right. The patient reported projectile vomiting for the previous five months, virtually daily for the first four months; the vomiting has decreased in the last two months as he consumes less food. His vomitus contains undigested food particles consumed more than 12 hours earlier. He has no history of hematemesis or melena. For the previous four months, the patient reported a lack of appetite and severe weight loss concerns. There is no previous history of peptic ulcer illness. The results of his routine laboratory investigation were within reference ranges. We conducted a double-contrast barium meal x-ray which showed a filling defect by the mass with proximal dilation and minimum contract on the first part of the duodenum (images unavailable-poor quality). Gastroscopy revealed a developing stomach bulge. The ulcer bled abundantly and showed widespread neovascularization with an ill-defined boundary. The stomach's angle shifted due to the enlarged and congested mucosa around the tumor. On the surface, it looked like stomach cancer. Figure 1 presents the endoscopic image of the ulcerated mass.

**Figure 2:**
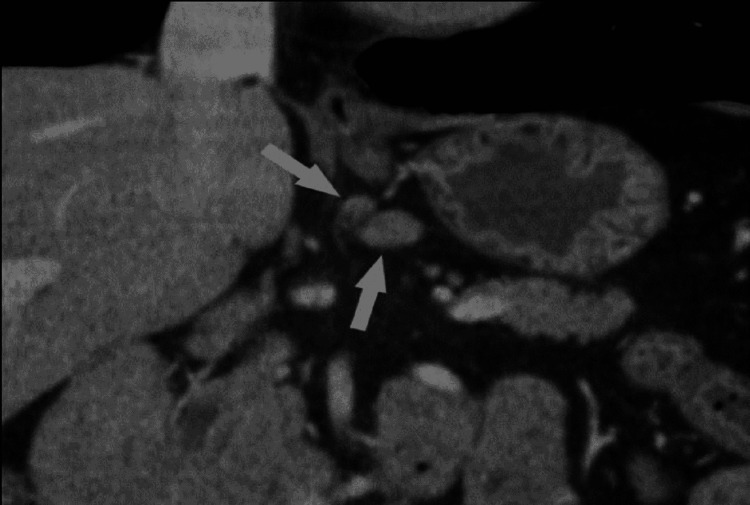
Abdominal computed tomography scan showing a mass in the stomach

A computed tomography (CT) scan of our patient's abdomen revealed a mass extending from the posterior wall of the greater curvature in the lower third of the stomach (Figure 2).

Due to the poor sensitivity of CT for detection of peritoneal metastasis and other visceral metastasis, our surgery team recommended a positron emission tomography (PET) scan, but financial restrictions prevented the use of PET. After a thorough examination with the relevant investigations, the patient was diagnosed with gastric outlet obstruction due to stomach carcinoma, so elective surgery was planned. Along with the gastric surgery, we resected the regional lymph node to improve prognosis and prevent recurrence and metastasis.

Next, we anastomosed the stump with the jejunum and conducted a Billroth II gastrojejunostomy with the restoration of the other organs as there was no distinct metastasis. We noted that the gross appearance was not indicative of adenocarcinoma of the stomach or any stomach lymphoma during surgery. Therefore, we sent the specimen for pathological evaluation due to a suspected diagnosis of RMS.

The tumor cells were spherical and ranged from lymphocyte-sized to six times larger than typical healthy cells. Eosinophilic cytoplasm and nucleoli were also seen in the fibrovascular septa after the intervention, and we noted a patchy eosinophilic pattern with necrotic zones. Tumor cells were stained with reddish bands resembling stripes, suggesting patchy necrosis. The sample included five to nine high-power fields (HPFs) associated with mitosis. Following a suspicion of RMS, a biopsy sample was submitted to a separate laboratory for RMS confirmation, which was not feasible in our facility. The sample was desmin-positive, and MyoD1 was highly expressed, confirming our primary epithelial RMS (ERMS) diagnosis of the stomach.

The patient's postoperative days were uneventful, and he recovered well. Fifteen days after admission, he was discharged home. Given the lack of postsurgical chemotherapy guidelines for RMS patients, he did not receive chemotherapy but was instructed to follow up regularly to monitor his condition.

## Discussion

RMS cells are uniformly eosinophilic with an elevated nuclear-cytoplasmic ratio. The cells are dispersed and exhibit a pulmonary alveolar pattern. There are several “nests” of tumor cells scattered throughout the tumor, formed by a network of fibrous septa [3]. RMS of the alveoli is the most common, comprising approximately 31% of all occurrences [4]. The extremities, trunk, and perianal regions are often affected, and adolescence is the most common age group.

RMS has four distinct subtypes recognized by the World Health Organization, with ERMS as the newest type. ERMS is akin to poorly differentiated malignancy or melanoma in that it has large, lobate hyperchromatic nuclei and multipolar mitotic patterns when studied under a microscope [5-7]. ERMS is distinct from other cancers because of aberrant cytokeratin positivity [8]. In the scientific literature, ERMS has been recorded just seven times [9]. A case series on RMS in Japan had seven cases (five men and two women aged 19 to 84; mean age, 56 years). Cancers appeared in the somatic soft tissue in four cases, tumors in organs appeared in two cases, and tumors in bone in one case. The mean tumor size was 10.5 cm (range, 3.5 cm to 15 cm) [10].

Stomach primary RMS is uncommon but fatal, and primary stomach ERMS has never been reported previously. Other ERMS-affected areas have similar histological features to our case. Most sarcomas include cytokeratin; however, they are also seen in certain carcinomas-the immune system's phenotype changes due to cell transformation and differentiation during ERMS development. The immune phenotypic change differentiates RMS from other gastric cancers, and MyoD1 is specific for myoglobin, which is specific for RMS. RMS is usually diagnosed via histological and electron microscopic findings.

The cytoplasm and nucleus of epithelioid RMS cancer cells, typically tiny to medium in size, do not have clear borders. Tumor cells may be arranged in various ways, including clustered, nested, or flaky. Malignancy might be indicated by necrotic patches and two or more mitotic figures in an HPF. Despite Syn, CD56, PGP9.5, and neuron-specific enolase (NSE), cancer cells do not express MyoD1, myoglobin, or desmin [11].

The origin of our patient’s RMS was most likely early-stage stomach epithelioid carcinoma of the lamina propria because the tumor lacks fibrous connective tissue and appears solid and nest-like in the histopathological laboratory report. The basophilic cytoplasm of tumor cells has a high concentration of melanin particles. Most of the nucleus is occupied by the massive, naturally eosinophilic nucleolus. HMB45 and MART-1, two tumor-specific markers, both show significant activity levels [12].

## Conclusions

In rare cases, the simple clinical presentation of gastric outlet obstruction might indicate a severe condition like RMS. An early diagnosis changes the management protocol. RMSs are common to the genitourinary and musculoskeletal systems but might appear in an unusual location like the stomach. This case highlights an extremely rare presentation of primary ERMS in the stomach. Information is limited regarding the laboratory investigations and management protocol for this condition, and clarity is needed regarding the use of chemotherapy after surgical correction.
